# Influence of Different Porosities of Titanium Meshes on Bone Neoformation: Pre-Clinical Animal Study with Microtomographic and Histomorphometric Evaluation

**DOI:** 10.3390/jfb14100485

**Published:** 2023-09-22

**Authors:** Rafael Mantovani, Ytalo Fernandes, Jonathan Meza-Mauricio, Danilo Reino, Laura Sanches Gonçalves, Luiz Gustavo Sousa, Adriana Luisa Almeida, Marcelo Faveri, Sergio Scombatti de Souza

**Affiliations:** 1 Department of Basic and Oral Biology, School of Dentistry of Ribeirao Preto, University of Sao Paulo, Ribeirao Preto 14040-904, SP, Brazil; rafaelvmantovani@hotmail.com (R.M.); sousalg@forp.usp.br (L.G.S.); 2 Department of Dentistry, Faculty Unicamps Goiânia, Rua 210, Setor Coimbra 386, Goiânia 74535-280, GO, Brazil; ytalo_odonto@usp.br; 3 Department of Periodontology, Universidad Cientifica del Sur, Calle Cantuarias 398, Lima 15048, Miraflores, Peru; emezam@cientifica.edu.pe; 4 Department of Oral and Maxillofacial Surgery and Periodontology, School of Dentistry of Ribeirao Preto, University of Sao Paulo, Av do Café, s/n., Ribeirao Preto 14040-904, SP, Brazil; danilomr2005@yahoo.com.br (D.R.); laurasg@usp.br (L.S.G.); aalmeida@forp.usp.br (A.L.A.); 5 Independent Researcher, Guarulhos 07072-000, SP, Brazil; mdfaveri@uol.com.br

**Keywords:** guided bone regeneration, titanium mesh, animal study, PTFE membrane

## Abstract

The aim of this study was to evaluate the influence of different types of porosity of titanium meshes on the bone neoformation process in critical defects surgically created in rat calvaria, by means of microtomographic and histomorphometric analyses. Defects of 5 mm in diameter were created in the calvaria of 36 rats, and the animals were randomly treated and divided into the following groups (6 animals per group): NCOG (negative control, only blood clot), TEMG (Polytetrafluoroethylene—PTFE—membrane), SPTMG (small pore titanium mesh), SPMMG (small pore mesh + PTFE), LPTMG (large pore titanium mesh), and LPMMG (large pore mesh + PTFE). After 60 days, the animals were sacrificed, and the bone tissue formed was evaluated with micro-CT and histomorphometry. The data were compared using an ANOVA followed by the Tukey post-test (*p* ≤ 0.05). The microtomographic results showed that the SPTMG group presented the highest numerical value for bone volume/total volume (22.24 ± 8.97), with statistically significant differences for all the other groups except LPTMG. Considering the histomorphometric evaluation, groups with only porous titanium meshes showed higher values compared to the groups that used the PTFE membrane and the negative control. The SPTMG group presented higher values in the parameters of area (0.44 mm^2^ ± 0.06), extension (1.19 mm^2^ ± 0.12), and percentage (7.56 ± 1.45%) of neoformed bone. It was concluded that titanium mesh with smaller pores showed better results and that the association of PTFE membranes with titanium meshes did not improve the outcomes, suggesting a correlation between mesh porosity and underlying bone repair.

## 1. Introduction

Dental implants are an important rehabilitative alternative to replacing missing teeth; however, successful installation of the implant is related to the quality and amount of bone in edentulous areas [1]. Tooth removal is often followed by a bone remodeling process, leading to a gradual reduction in horizontal and vertical bone ridge height [2]. Bone resorption primarily occurs in the buccal aspect and increases over time [3], and these changes can negatively affect the esthetic results of a final rehabilitation, regardless of the type of dental rehabilitation performed [4]. If adequate three-dimensional (3D) positioning of implants cannot be achieved in the residual bone, a guided bone regeneration (GBR) procedure should be performed [5]. 

GBR has been defined as the use of barrier membranes, either absorbable or not, to exclude certain cell types, such as epithelium and connective tissue, and promote the growth of cells of the osteoblastic lineage [6]. GBR is often combined with bone grafting procedures. Until now, the ideal mechanical barrier for GBR has been described in studies aimed at analyzing factors such as stability, occlusivity, peripheral sealing between barrier and bone tissue, ideal pore size, blood supply required, and providing proliferation of osteoprogenitor cells [7]. Titanium mesh has been widely used as a barrier and can be adapted to keep volume during the healing period without graft compression by the flap [8]. Several studies have shown satisfactory results for bone reconstructions with titanium meshes [9,10,11].

The design of barriers manufactured for GBR has generally included barrier perforation with the intention of optimizing conditions for bone formation [12]. To date, the influence of macroporosity remains controversial [13,14,15]. Previous studies have suggested that the titanium mesh porosity plays an important role in establishing the supplement of osteogenic cells, growth factors, and blood supply from the periosteal tissue overlying the barrier [13,14]. The porosity is also important to the mechanical properties of biomaterials [16].

However, it has recently been demonstrated that bone augmentation regularly also occurs beneath totally occlusive barriers [17]. However, it has not been revealed whether a perforated barrier could influence the quantity and quality of the augmented bone tissue as well as the velocity of its formation. On the other hand, some studies have suggested that larger diameter pore size allows new bone angiogenesis and better nutrient diffusion, while occlusive meshes may limit the neovascularization process and also restrict fibrous connective tissue invasion [9,18].

Therefore, the aim of this study was to perform microtomographic and histomorphometric evaluations to determine the influence of two different porosity sizes of titanium meshes, covered or not with a Polytetrafluoroethylene (PTFE) membrane, on the bone formation process in critical bone defects surgically created in rat calvaria.

## 2. Materials and Methods

### 2.1. Ethical Aspects and Financial Support

This research project was sent for evaluation to the Ethics Committee on Animal Experimentation of the School of Dentistry of Ribeirao Preto, University of São Paulo, and was duly approved and registered with protocol number 2018.1.829.68.6. ARRIVE guidelines were consulted in reporting this study [19].

### 2.2. Sample Characterization

Forty-eight 3-month-old Sprague Dawley rats with an average weight of 250 g–300 g from the University of São Paulo, Ribeirão Preto central laboratory, were used in this study. The animals were kept in appropriate plastic boxes with food and water ad libitum before and during the experimental period and remained in the vivarium of the School of Dentistry of Ribeirão Preto in a 12 h cycle environment of light and temperature between 23.5 °C and 24.5 °C.

#### Experimental Groups

Using a computer-generated random sequence, 48 animals were randomly allocated into six experimental groups:

Group 1 (NCOG): Negative control group, in which the bone defect was filled only with blood clot (*n* = 8).

Group 2 (TEMG): PTFE membrane group, in which the bone defect was filled with blood clot and covered with PTFE membrane (Surgitime PTFE, Bionnovation Biomedical Bauru, SP, Brazil) (*n* = 8).

Group 3 (SPTMG): Small pore titanium mesh group, in which the bone defect was filled with blood clot and covered with small pore titanium mesh, with a thickness of 0.04 mm and a pore size of 154.4 µm (Surgitime Titanium 34 × 25 × 0.04 mm with hole of 0.15 mm, Bionnovation Biomedical, Bauru, SP, Brazil) (*n* = 8).

Group 4 (SPMMG): Small pore mesh + membrane group, in which the bone defect was filled with blood clot and covered with the small pore titanium mesh (0.04 mm thickness and 154.4 µm pore size) + PTFE membrane over the mesh (*n* = 8).

Group 5 (LPTMG): Large pore titanium mesh group, in which the bone defect was filled with blood clot and covered with large pore titanium mesh with 0.04 mm thickness and 850 µm pore size (Surgitime Titanium 34 × 25 × 0.04 mm with hole of 0.85 mm, Bionnovation Biomedical, Brazil) (*n* = 8).

Group 6 (LPMMG): Large pore mesh + membrane group, in which the bone defect was filled with blood clot and covered by the large pore titanium mesh (0.04 mm thickness and 850 µm pore size) + PTFE membrane over the mesh (*n* = 8).

The meshes used in this study were manufactured through the chemical machining process. This process consists of removing material from specific areas, through the corrosion of the substrate by a strong chemical reaction through a reagent substance, under controlled conditions.

### 2.3. Surgical Procedure

**Figure 1:**
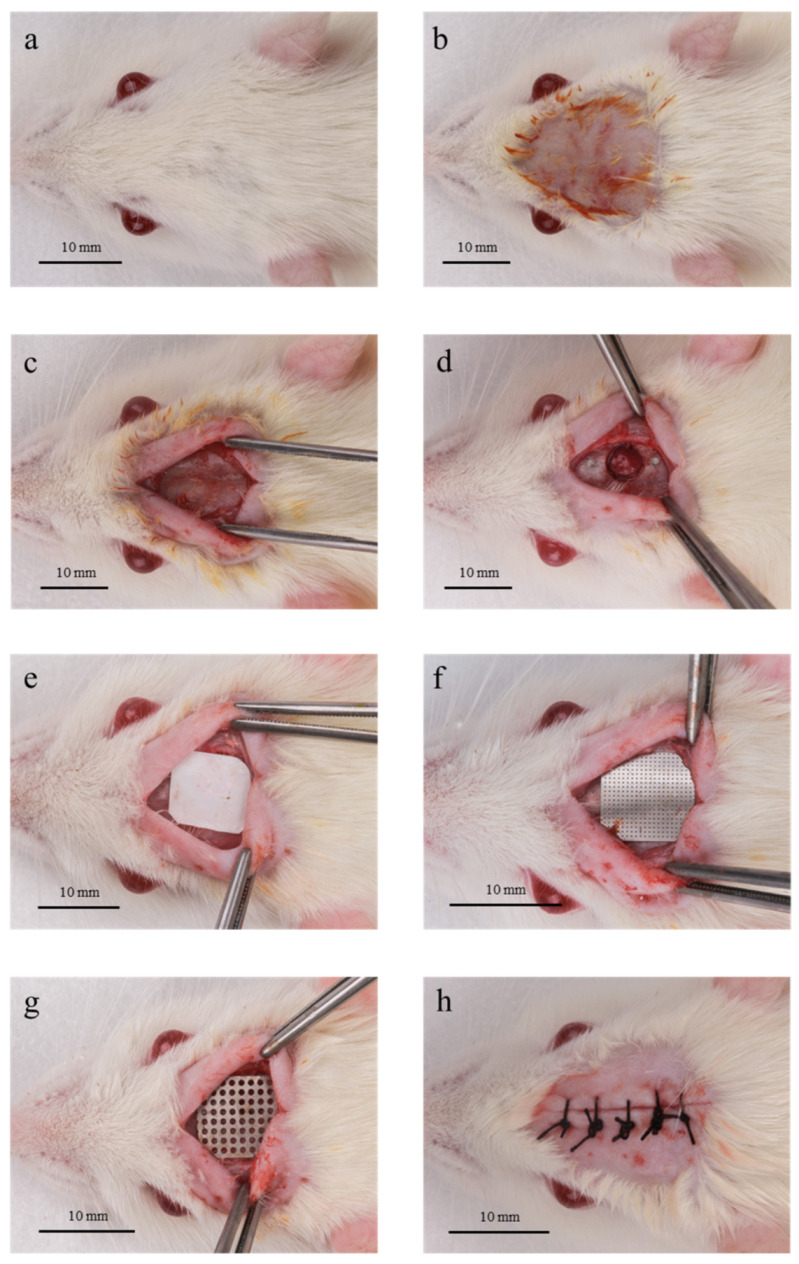
Surgical sequence. (**a**): animal anesthetized and in position; (**b**): tricotomy and antisepsis performed; (**c**): incision made and flap elevated, exposing the bone tissue; (**d**): critical size defect (5 mm in diameter) made and markings filled with amalgam marking the center line of the defect; (**e**): Group 2, surgical defect covered by a PTFE membrane; (**f**): Group 3, surgical defect covered with small-pored titanium mesh; (**g**) Group 4, surgical defect covered with large pore titanium mesh; (**h**): final aspect after flap suturing.

Animals received general anesthesia, obtained by the association of 2% (2 mg/mL) of xylazine hydrochloride (Rompum^®^—Bayer Saúde Animal, São Paulo, SP, Brazil) and 10% (10 mg/mL) of ketamine hydrochloride (Dopalen^®^—Ceva Saúde Animal Ltda., Paulínia, SP, Brazil), via intramuscular injection at concentrations of 10 mg/Kg and 80 mg/Kg, respectively (Figure 1a). Subsequently, trichotomy and antisepsis of the dorsal region of the skull was performed with a 1% povidone-iodine solution (Figure 1b). A linear incision was made to access the bone tissue, followed by full-thickness flap elevation. (Figure 1c). Subsequently, one standardized critical size defect of 5 mm in diameter was made at the center of the parietal bone using a trephine (Broca Trefina 5 mm, Harte Instrumentos Cirúrgicos, Ribeirão Preto, SP, Brazil). The trephine burr was used under constant irrigation with 0.9% saline to prepare the defect without damaging the inner dura mater of the cranial bone.

To facilitate identification of the central region of the original bone defect in the laboratory and histomorphometric procedures, two 2 mm markings, one anterior and one posterior to the critical size defect, were made and filled with amalgam (Figure 1d). Diamond drills were used (#2200, KG Sorensen^®^, Cotia, SP, Brazil) for this procedure under constant irrigation with sterile saline solution (0.9%).

Blood clots were used to fill all the defects. In Group 1 animals, no additional biomaterial was applied over the defects. In Group 2 animals, a PTFE membrane was adapted over the surgical defect (Figure 1e). In Group 3, the defect was covered with small-pored titanium mesh (Figure 1f). In Group 4, the bone defect was covered with small pore titanium mesh + PTFE membrane. In Group 5, the defect was covered with large pore titanium mesh (Figure 1g). In Group 6, the bone defect was covered with large-pored titanium mesh + PTFE membrane. The dimensions of the materials used to cover the defects (titanium meshes and PTFE membranes) were standardized at 10 mm wide by 10 mm high. Primary closure of the tissues was accomplished using non-absorbable sutures (Seda Ethicon 5.0, Johnson Prod., São José dos Campos, Brazil) (Figure 1h).

After surgery, the animals received a single intramuscular antibiotic dose of 24,000 IU/kg penicillin G-benzathine (Pentabiótico Veterinário Pequeno Porte, Fort Dodge Animal Health^®^, Campinas, SP, Brazil) at a dose of 0.01 mL per 100 g of body weight and the anti-inflammatory Banamine^®^ via intramuscular injection 1 mg/mL, at a dose of 2 mL/Kg (Injetável Pet—Schering-Plough, Cotia, SP, Brazil). The operated animals remained under constant observation and were placed in autoclavable polypropylene boxes (3 animals per box) for anesthetic recovery. After 60 days, the animals were euthanized and block calvaria bone biopsy specimens, including the membranes and meshes, were obtained for subsequent 3D micro-computed tomography (micro-CT) and histomorphometric analysis.

### 2.4. Micro-Computed Tomography Analysis

Unprocessed bone biopsy specimens were imaged and analyzed using micro-CT (Skyscan 1172, Bruker, Kontich, Belgium) at 50 KV and a resolution of 1 µm. An experienced examiner performed the analyses using CT-Analyser^®^ v.1.13.5.1+ software (Bruker, Kontich, Belgium).

**Figure 2:**
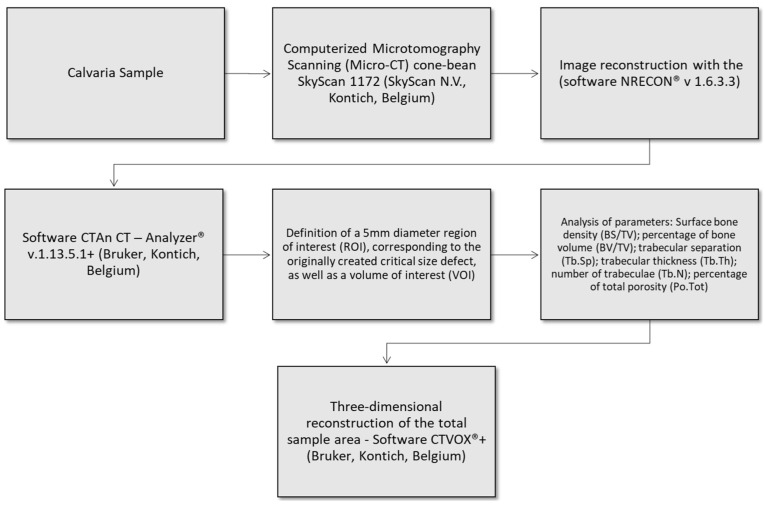
Flowchart of micro-CT evaluation sequence.

The following tomographic parameters were evaluated: bone surface density (BS/TV), corresponding to the ratio between the area of the bone surface and the volume of the region of interest, expressed in 1/mm (ratio of the segmented bone surface to the total volume of the region of interest); percentage of bone volume (BV/TV), which is the ratio of bone volume to total volume, expressed in % (bone volume fraction, ratio of the segmented bone volume to the total volume of the region of interest); trabecular separation (Tb.Sp), expressed in mm (mean distance between trabeculae, assessed using direct 3D methods); trabecular thickness (Tb.Th), expressed in mm (mean thickness of trabeculae, assessed using direct 3D methods); number of trabeculae (Tb.N), expressed in 1/mm (measure of the average number of trabeculae per unit length); and percentage of total porosity (Po.Tot), expressed in % (measure of the percentage of bone porosity in the region of interest). Figure 2 shows a flowchart explaining how the quantification of these parameters was performed.

### 2.5. Histological Processing

After euthanasia, block calvarial bone pieces were fixed in 4% neutral formalin for 10 days. The pieces were then transferred to a 70% ethanol solution until processing. Dehydration of the specimens was performed in an ethanol gradient at increasing concentrations (70%, 95%, and 100% solutions).

All parts were infiltrated and included in LR White resin (London Resin Company, Berkshire, UK). Subsequently, the parts were subjected to the micro-wear system (Exakt, Germany) using the hard tissue sectioning technique described by Donath and Breuner, 1982 [20], in order to obtain slides of approximately 50 to 80 μm. The sections were mounted on histological slides for analysis and stained with Stevenel’s blue and Alizarin Red S.

### 2.6. Histomorphometry

**Figure 3:**
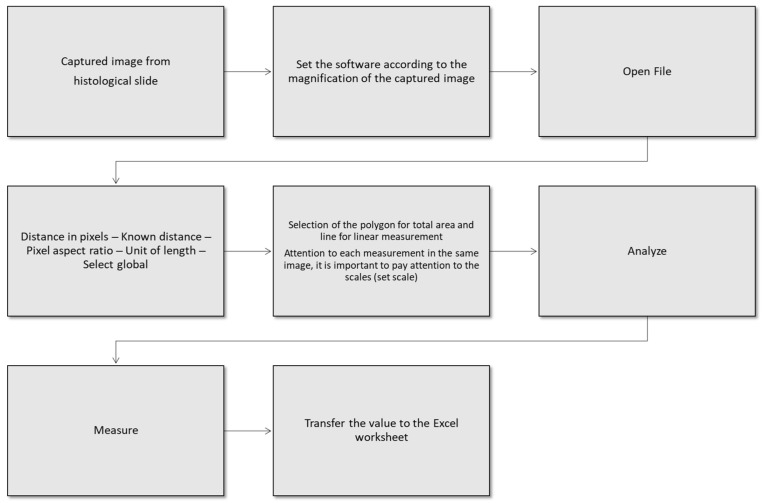
Flowchart of bone quantification procedures.

A 50–80 µm thick longitudinal histological section of the rat calvaria was captured using a Leica DC 300F video camera (Leica Microsystems GmbH, Nussloch, Germany) coupled to a Leica MZFL III stereomicroscope (Leica Microsystems GmbH, Nussloch, Germany). The images were analyzed using Image J software (National Institutes of Health, Bethesda, MD, USA) for the determination of the linear amount and area of bone formation. Figure 3 shows a flowchart that explains the bone quantification with Image J.

**Figure 4:**
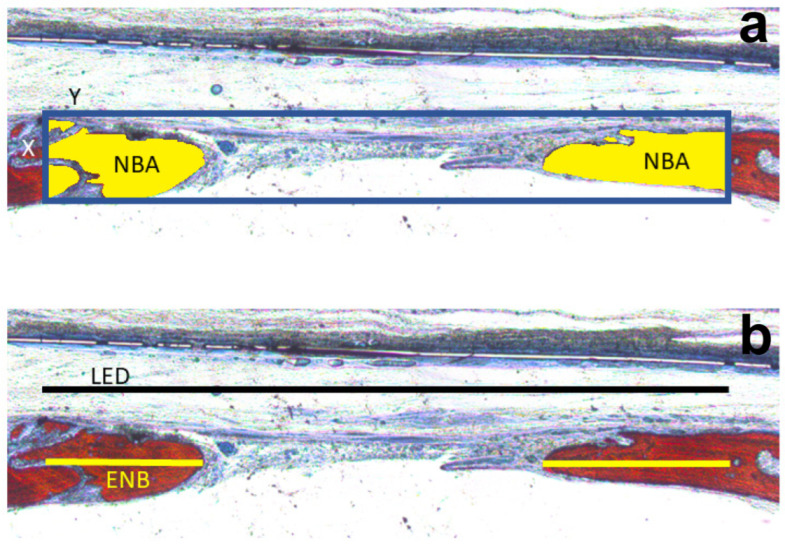
Schematics of the area and linear histomorphometric measurements. (**a**): TA = total area of the defect (blue box in the figure), determined by the area in mm2 resulting from the multiplication of the measurements of defect width (Y) by effect height (X); NBA (in yellow) = area of new bone, measured inside the total area, in mm^2^, as the neoformed bone in the defect from its edges. LED = total linear extension of the defect, in mm; (**b**) ENB = extension of new bone, in mm, neoformed into the defect from its borders. The NBA in percent was calculated using the formula NBA/TA; the ENB in percent was calculated using the formula ENB/LED.

The following parameters were evaluated: total area (TA) in mm^2^ of the originally created defect; neoformed bone area (NBA) within the TA, calculated as a percentage and also measured as a percentage of the TA; linear extent of the created surgical defect (LED), having as limits the delimited extremities for the measurement of the TA and extent of neoformed bone (ENB), also calculated and expressed in mm (Figure 4).

### 2.7. Statistical Analysis

The statistical analyses were performed with the GraphPad Prism version 5.0 statistical program. For comparisons between groups, we used Simple Analysis of Variance (one-way ANOVA) followed by the Tukey post-test to identify samples that are statistically different from each other. A significance level of 5% was used for all the statistical analyses.

## 3. Results

### 3.1. Micro-Computed Tomography Analysis

**Figure 5:**
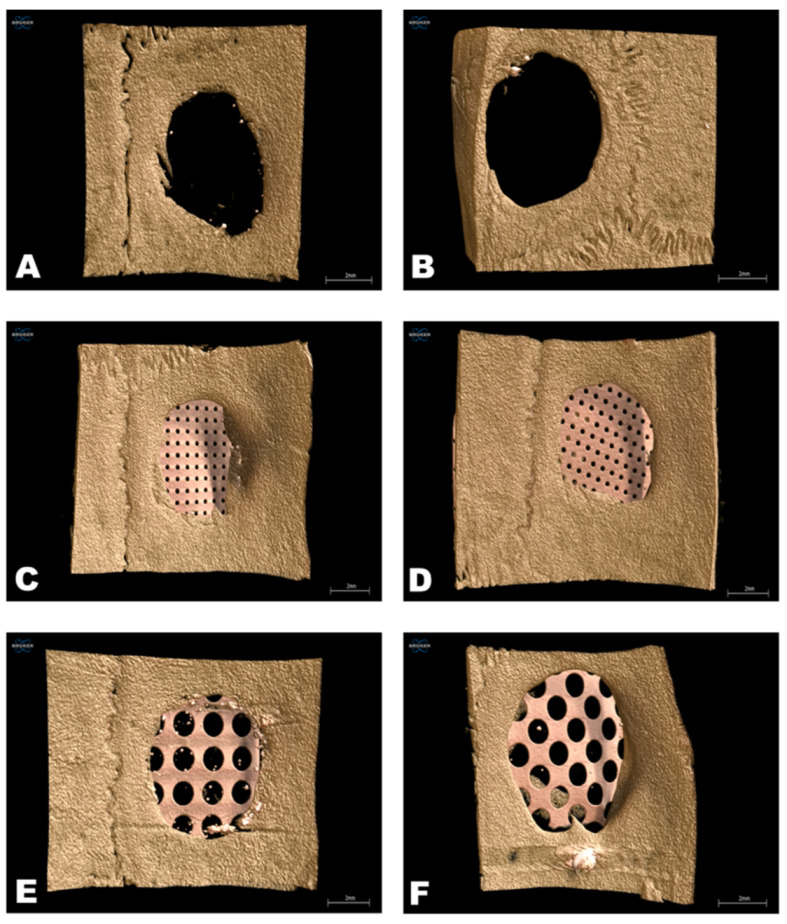
Micro-CT analysis—three-dimensional reconstructions of the defects. (**A**): Group 1 (NCOG), negative control (clot); (**B**): Group 2 (TEMG), clot + PTFE membrane; (**C**): Group 3 (SPTMG), clot + small pore titanium mesh; (**D**): Group 4 (SPMMG), clot + small pore titanium mesh + PTFE membrane; (**E**): Group 5 (LPTMG), clot + large pore titanium mesh; and (**F**): Group 6 (LPMMG), clot + large pore titanium mesh + PTFE membrane.

The micro-CT analysis was performed by one experienced and blinded examiner who evaluated the following parameters: BS/TV, BV/TV, Tb.SP, Tb.Th, Tb.N, and Po.Tot. Figure 5 shows representative 3D reconstructions of each group analyzed.

#### 3.1.1. Surface Bone Density (BS/TV)

**Figure 6:**
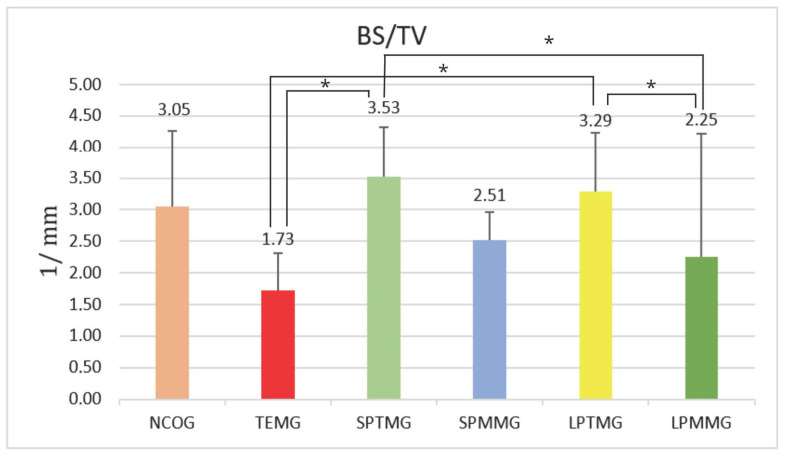
Mean ± standard deviation of surface bone density (BS/TV) values. The * sign means the presence of statistically significant differences between groups (*p* < 0.05).

The results of the BS/TV (Figure 6) showed that Group 3 (SPTMG) had better outcomes, with statistically significant differences compared to Group 2 (TEMG) (1.75 ± 0.59) (*p* = 0.004) and Group 6 (LPMMG) (2.25 ± 1.96) (*p* = 0.024). Additionally, statistically significant differences were found between Group 5 (LPTMG) (3.29 ± 0.94) and Group 2 (TEMG) (1.73 ± 0.59) (*p* = 0.008), as well as between Group 5 (LPTMG) (3.29 ± 0.94) and Group 6 (LPMMG) (2.25 ± 1.96) (*p* = 0.045).

#### 3.1.2. Percentage of Bone Volume (BV/TV)

**Figure 7:**
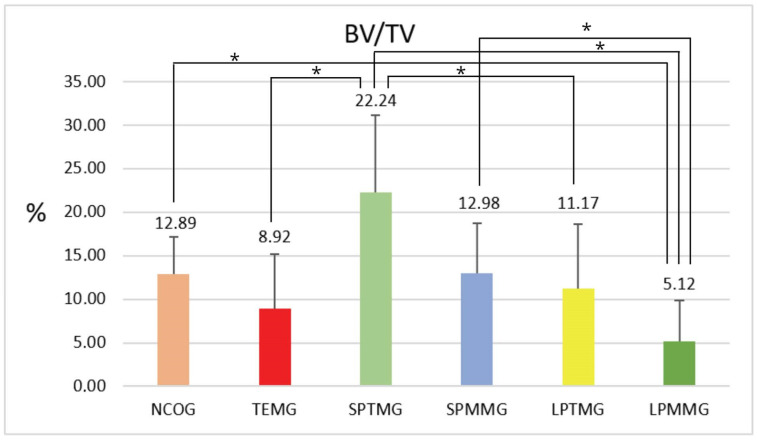
Mean ± standard deviation of the percentage of bone volume (BV/TV) values. The * sign means the presence of statistically significant differences between groups (*p* < 0.05).

There were statistically significant differences between Groups 1 (NCOG) (12.89 ± 4.32) and 6 (LPMMG) (5.12 ± 4.71), with *p* = 0.032. Group 3 (SPTMG) had the highest mean value among all the experimental groups (22.24 ± 8.97), with statistically significant differences for Groups 2 (TEMG) (8.92 ± 6.29), *p* = 0.008, 5 (LPTMG) (11.17 ± 7.50), *p* = 0.026, and 6 (LPMMG) (5.12 ± 4.71), *p* = 0.0003. There were also statistically significant differences between Groups 4 (SPMMG) (12.98 ± 5.72) and 6 (LPMMG) (5.12 ± 4.71), *p* = 0.039 (Figure 7).

#### 3.1.3. Trabecular Separation (Tb.Sp)

**Figure 8:**
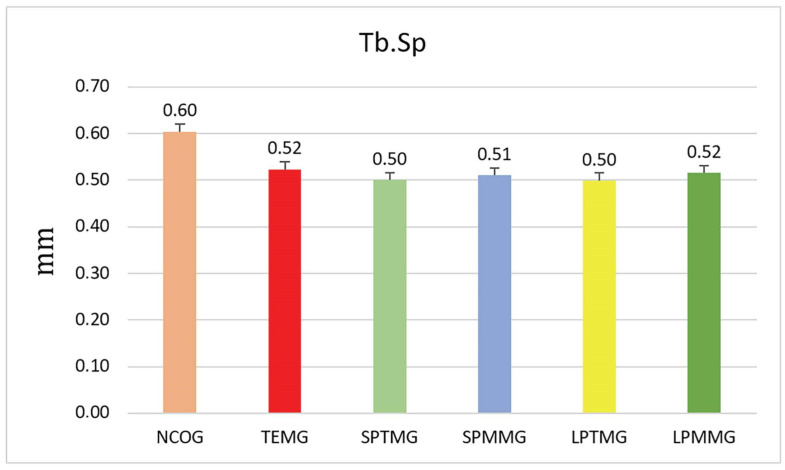
Mean ± standard deviation of trabecular separation (Tb.Sp) values. There were no statistically significant differences between the groups (*p* = 0.21).

There were no statistically significant differences between groups (*p* = 0.21). Group 1 (NCOG) presented the highest numerical mean among all the groups analyzed (0.60 ± 0.27), while Groups 2 (SPTMG) (0.50 ± 0.03) and 5 (LPTMG) (0.50 ± 0.02) presented the lowest numerical means (Figure 8).

#### 3.1.4. Trabecular Thickness (Tb.Th)

**Figure 9:**
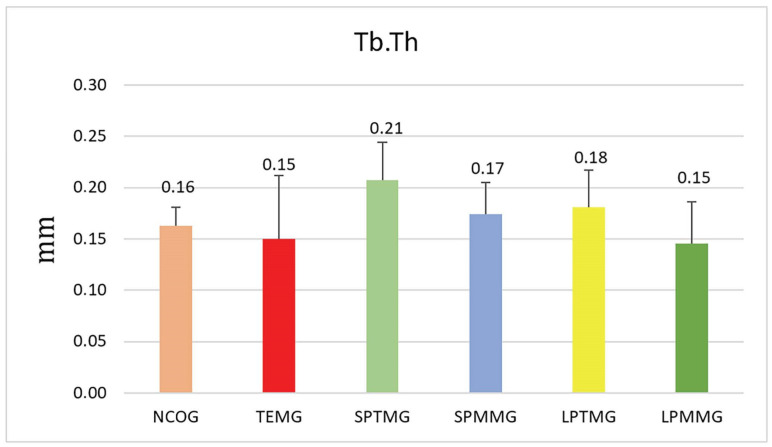
Mean ± standard deviation of trabecular thickness values (Tb.Th). There were no statistically significant differences between groups (*p* = 0.12).

Group 3 (SPTMG) showed the highest numerical value (0.21 ± 0.04), and Groups 1 (TEMG) (0.15 ± 0.06) and 6 (LPMMG) (0.15 ± 0.04) had the lowest values. However, there were no statistically significant differences among the groups (*p* = 0.12) (Figure 9).

#### 3.1.5. Number of Trabeculae (Tb.N)

**Figure 10:**
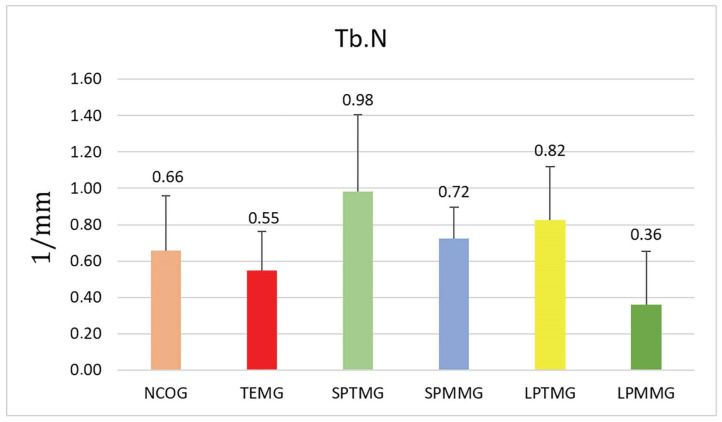
Mean ± standard deviation of the number of trabeculae values. There were no statistically significant differences between the groups (*p* = 0.07).

Group 3 (SPTMG) showed the highest numerical value (0.98 ± 0.42) and Group 6 (LPMMG) the lowest value (0.36 ± 0.29). However, there were no statistically significant differences among the groups (*p* = 0.07) (Figure 10).

#### 3.1.6. Total Porosity (Po.Tot)

**Figure 11:**
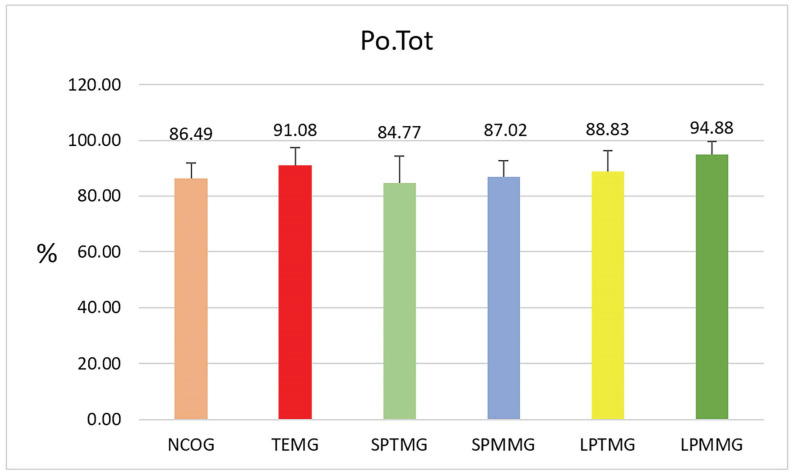
Mean ± standard deviation of the total porosity values. There were no statistically significant differences between the groups (*p* = 0.10).

There were no statistically significant differences between the groups (*p* = 0.10). The highest numerical value of porosity was obtained by Group 6 (LPMMG) (94.88 ± 4.71%), while the lowest value was presented by Group 3 (SPTMG) (84.77 ± 9.47%) (Figure 11).

## 4. Descriptive Histological Analysis

**Figure 12:**
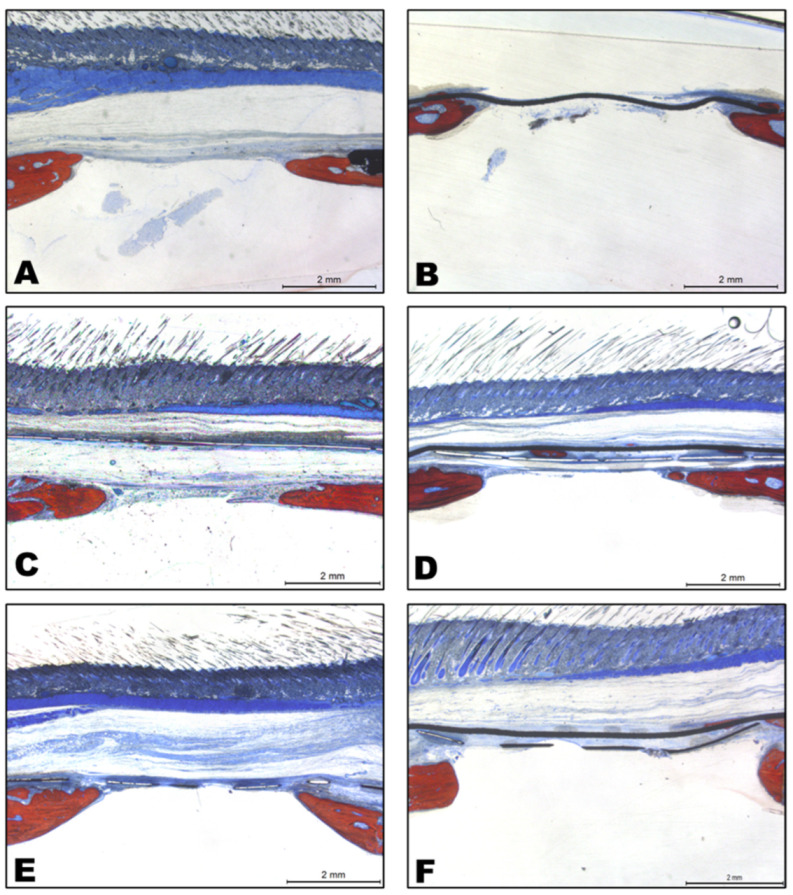
Representative panoramic histological images of the experimental groups. (**A**): Group 1 (NCOG), showing small bone neoformation from the edges of the defect, and a fibrous layer in continuity with it, suggesting the formation of a pseudo periosteum; (**B**): Group 2 (TEMG), showing limited bone formation from the edges of the defect and no fibrous tissue under the membrane; (**C**): Group 3 (SPTMG), showing the most bone formation from the edges of the defect as well as the presence of fibrous tissue under the mesh; (**D**): Group 4 (SPMMG), showing bone neoformation between the membrane and mesh, disconnected from the edges of the original bone defect; (**E**) Group 5 (LPTMG), showing bone formation from the edges and the absence of fibrous tissue under the mesh; (**F**) Group 6 (LPMMG), showing limited bone neoformation and the absence of substantial fibrous tissue under the mesh + membrane assembly.

The panoramic images of all the groups analyzed are represented in Figure 12. To a greater or lesser extent, all the groups presented centripetal bone neoformation, starting at the edges of the defect. The groups with the PTFE membrane plus the titanium meshes showed less bone formation than the respective groups without the membrane, but the groups with the barrier did not allow connective tissue migration toward the defect. Although Group 3 (SPTMG) showed the highest values of bone neoformation histomorphometrically, connective tissue migration passing the mesh toward the defect was observed (Figure 12C), a finding that was not repeated in the Group 5 (LPTMG) specimens (Figure 12E). Some Group 4 (SPMMG) specimens (small pore mesh + PTFE membrane) showed some bone neoformation between the membrane and mesh, disconnected from the edges of the bone defect (Figure 12D).

### 4.1. Histomorphometry Analysis

#### 4.1.1. Total Area Bone Defect (TA)

**Figure 13:**
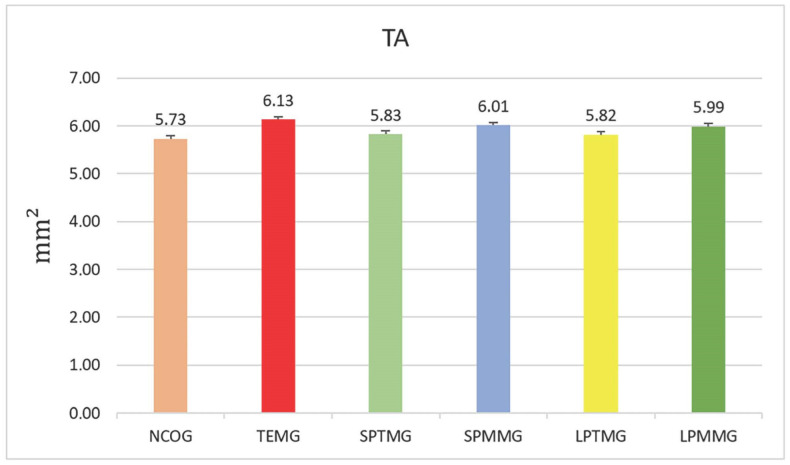
Total area of the bone defect (mm^2^). There were no statistically significant differences between the groups (*p* = 0.07), showing uniformity in the methodology for making the experimental bone defect.

The TA measurement did not reveal any statistically significant differences among the experimental groups (*p* = 0.07), indicating that the defects were properly standardized (Figure 13).

#### 4.1.2. Neoformed Bone Area (NBA)

**Figure 14:**
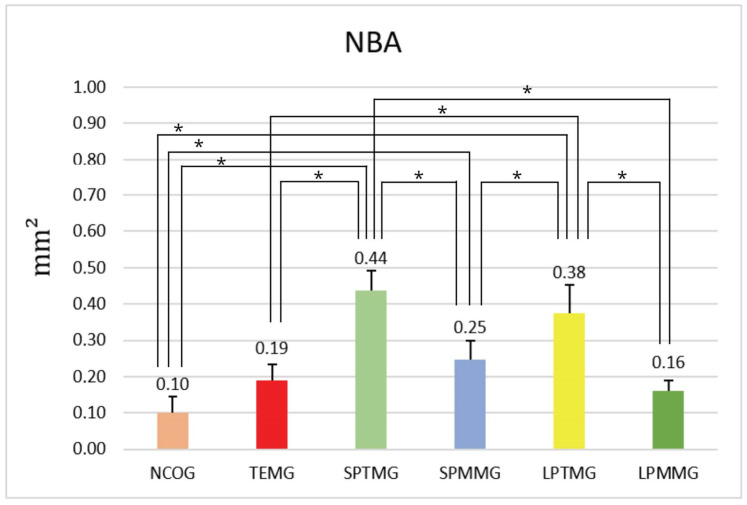
Mean ± standard deviation of the values of neoformed bone area, in mm^2^. * indicates the presence of statistically significant differences between groups (*p* < 0.05).

The highest numerical value of NBA was presented by Group 3 (SPTMG), closely followed by Group 5 (LPTMG). There were statistically significant differences between Group 1 (NCOG) (0.10 ± 0.04) and Group 3 (SPTMG) (0.44 ± 0.06), *p* < 0.01; Group 1 (NCOG) and Group 4 (SPMMG), *p* < 0.01; as well as between Group 1 (NCOG) and Group 5 (LPTMG), *p* < 0.01. Group 2 (TEMG) (0.19 ± 0.04) showed statistically significant differences compared to Groups 3 (SPTMG) (0.44 ± 0.06) and 5 (LPTMG) (0.38 ± 0.08), *p* < 0.01. There were also statistically significant differences between Groups 3 (SPTMG) (0.44 ± 0.06) and 4 (SPMMG) (0.25 ± 0.05), *p* < 0.01, and between Groups 3 (SPTMG) (0.44 ± 0.06) and 6 (LPMMG) (0.16 ± 0.03), *p* < 0.01. Group 4 (SPMMG) (0.25 ± 0.05) versus Group 5 (LPTMG) (0.38 ± 0.08) also showed statistically significant differences, *p* < 0.001. Finally, there were also statistically significant differences between Groups 5 and 6 with *p* < 0.01 (Figure 14).

#### 4.1.3. Percentage of Neoformed Bone Area (% NBA)

**Figure 15:**
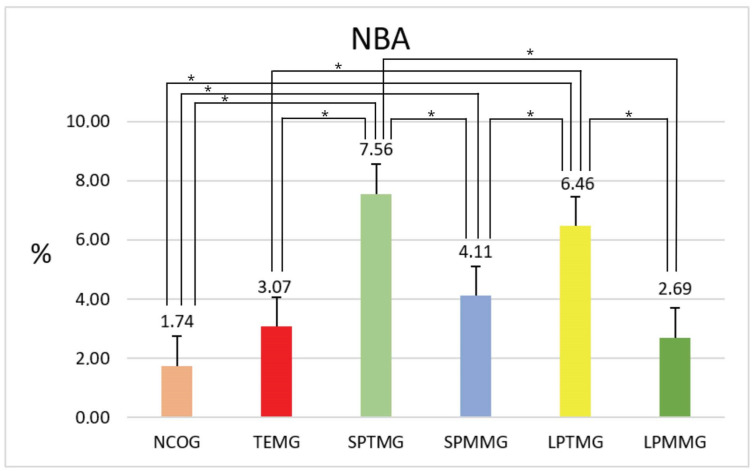
Mean ± standard deviation of the values of percentage of neoformed bone area, calculated as a percentage of TA. * indicates the presence of statistically significant differences between groups (*p* < 0.05).

The highest percentage value of NBA was presented by Group 3 (SPTMG) (7.56 ± 1.45%), with statistically significant differences for all other experimental groups (Group 1 (NCOG) = 1.74 ± 0.79, *p* < 0.01; Group 2 (TEMG) = 3.07 ± 0.66, *p* < 0.01; Group 4 (SPMMG) = 4.11 ± 0.84, *p* < 0.01; and Group 6 (LPMMG) = 2.69 ± 0.43, *p* < 0.01), with the exception of Group 5 (LPTMG) (6.46 ± 1.40). The latter group also showed the second highest numerical value for this parameter, with statistically significant differences compared to Groups 1 (NCOG), 2 (TEMG), 3 (SPTMG), and 6 (LPMMG) (Figure 15).

#### 4.1.4. Linear Extension of Defect

**Figure 16:**
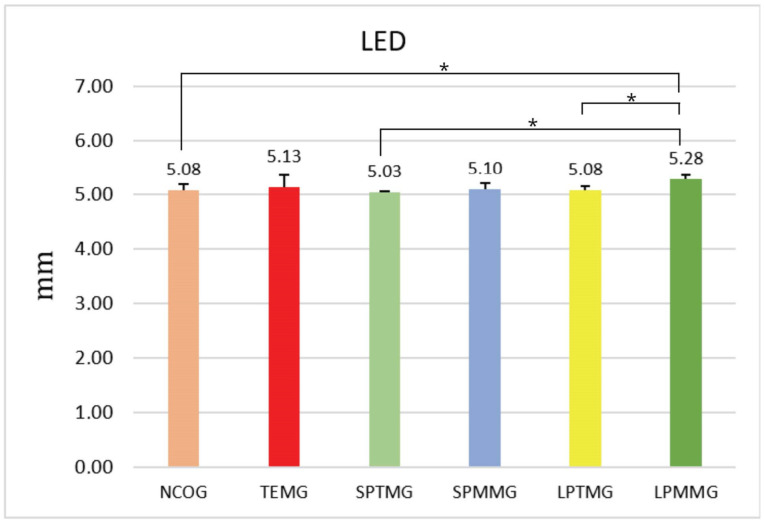
Mean ± standard deviation of the linear extension values, calculated in mm. * indicates the presence of statistically significant differences between groups (*p* < 0.05).

In the analysis of the linear extension of the surgical defect created, there were statistically significant differences between the following experimental groups: Group 1 (NCOG) (5.08 ± 0.12) versus 6 (LPMMG) (5.28 ± 0.07), *p* < 0.05; Group 3 (SPTMG) (5.03 ± 0.03) versus 6, *p* < 0.01; and Group 5 (LPTMG) (5.08 ± 0.07) versus 6 (LPMMG), *p* < 0.05 (Figure 16).

#### 4.1.5. Extent of Neoformed Bone (ENB)

**Figure 17:**
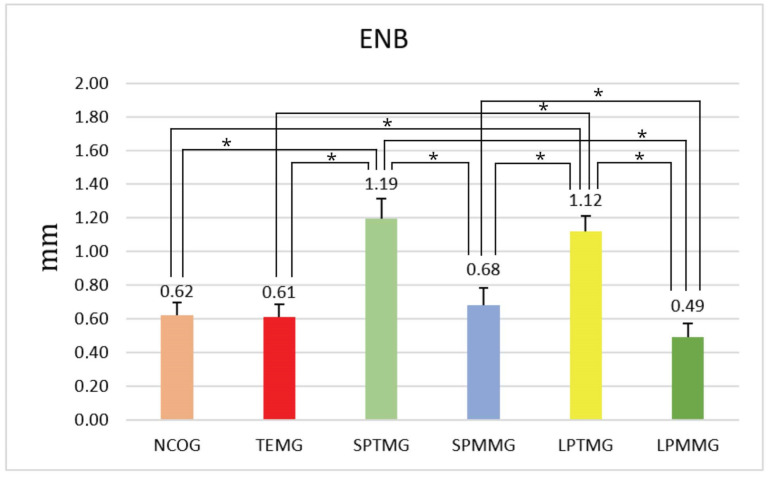
Mean ± standard deviation of the extent of neoformed bone values, calculated in mm. * indicates the presence of statistically significant differences between groups (*p* < 0.05).

Evaluation of the extent of neoformed bone in millimeters showed statistically significant differences between Group 1 (NCOG) (0.62 ± 0.08) and Groups 3 (SPTMG) (1.19 ± 0.12) and 5 (LPTMG) (1.12 ± 0.09), *p* < 0.01. There were also statistically significant differences between Groups 2 (TEMG) (0.61 ± 0.08) and 3 (SPTMG) (1.19 ± 0.12) (*p* < 0.01). Group 3 (SPTMG) (1.19 ± 0.12) showed statistically significant differences in relation to Groups 4 (SPMMG) (0.68 ± 0.10) and 6 (LPMMG) (0.49 ± 0.08) (for both, *p* < 0.01). There were also statistically significant differences between Groups 4 (SPMMG) and 5 (LPTMG), *p* < 0.01, and 4 (SPMMG) and 6 (LPMMG) (0.49 ± 0.08), *p* < 0.05. Finally, Groups 5 (LPTMG) and 6 (LPMMG) also showed statistically significant differences between them, with *p* < 0.01 (Figure 17).

#### 4.1.6. Percentage of Extension of Neoformed Bone

**Figure 18:**
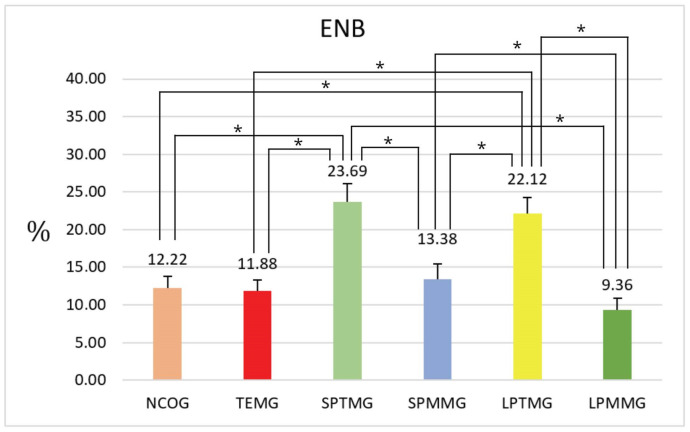
Mean ± standard deviation of extension neoformed bone values, calculated as a percentage. * indicates the presence of statistically significant differences between groups (*p* < 0.05).

The highest percentage value of ENB was presented by Group 3 (SPTMG) (23.69 ± 2.42), with statistically significant differences for all other experimental groups (Group 1 (NCOG) = 12.22 ± 1.58% *p* < 0.01; Group 2 (TEMG) = 11.88 ± 1.39, *p* < 0.01; Group 4 (SPMMG) = 13.38 ± 2.05, *p* < 0.01; Group 6 (LPMMG) = 9.36 ± 1.49, *p* < 0.01), with the exception of Group 5 (LPTMG) (22.12 ± 2.13). The latter group also showed the second highest numerical value for this parameter, with statistically significant differences in relation to the Groups 1 (NCOG), 2 (TEMG), 4 (SPMMG), and 6 (LPMMG) (Figure 18).

## 5. Discussion

The reconstruction of atrophic ridges is increasingly used in implantology to allow for the placement of dental implants with adequate dimensions in a three-dimensionally correct position. In this context, the use of a titanium mesh associated with particulate bone substitutes and/or autogenous grafts has proven to be an effective technique with adequate clinical outcomes [8,21,22,23].

In the present study, microtomographic and histomorphometric evaluations were performed to determine the influence of two different porosity sizes of titanium meshes on the bone neoformation process in critical bone defects surgically created in rat calvaria. The meshes evaluated contained pores of 154.4 µm or 850.0 µm and were or were not covered by a PTFE membrane occlusive to epithelial and connective tissue cells. Although the GBR technique advocates the use of occlusive barriers for undesirable tissues (epithelium and connective) to prevent their migration to the bone defect, the literature shows that a certain degree of porosity above the cellular size parameters of these tissues allows the migration of soft tissues but does not prevent bone neoformation [11,16,24].

The literature evaluating the differences between barrier porosities using microtomography is still scarce. In 2014, Rakhmatia and coworkers evaluated the ideal thickness and porosity of new titanium meshes in order to improve bone gain and prevent soft tissue growth and mesh exposure. Six types of new titanium meshes with different thicknesses (from 20 to 100 μm) and pore amounts (12 pores or multiple pores), together with three commercially available membranes, were used to cover surgically created calvaria defects in 100 rats divided into 20 groups [9]. The greatest bone volume was observed in 100 µm thick membranes with larger pores, although these membranes promoted bone growth with lower mineral density. According to the authors, membrane porosity is an essential factor for GBR, mainly in the initial healing period; nonetheless, the bone volume obtained was about the same. These results partially agree with those of the present study, in which the porosities present in the titanium meshes affected the qualitative parameters of the microtomographic evaluation. However, in our study, the 3D bone density (BV/TV) was higher in the groups with porosities contrary to the study by Rakhmatia [9].

Yanamoto and coworkers evaluated the effects of mechanical barrier permeability in a rat model of calvarial guided bone augmentation, using radiological and histological analyses. The calvaria of 20 rats were exposed, and one of four types of plastic caps was randomly placed (an occlusive cylindrical plastic cap, an open nonocclusive plastic cap, a plastic cap with three holes, and a plastic cap with four holes). The animals were euthanized after 6 and 12 weeks (10 animals at each time), and the neoformed bone inside the plastic capsules was evaluated using microtomography (measured after 4, 8, and 12 weeks) and histological analysis. The results of 3D bone neoformation, measured in mm^3^ using micro-CT, showed that the values decreased as the permeability of the barrier increased in all three observation periods. Volumes in the open group were consistently lower than those in the other three groups at all times, although with statistically significant differences only after 8 and 12 weeks [13]. In the present study, we did not evaluate bone formation quantitatively using micro-CT but qualitatively. Our results agree with those of Yamamoto et al. [13], as the groups with perforated meshes (Groups 3 (SPTMG) and 5 (LPTMG)) showed the highest values of surface bone density (BS/TV) and 3D bone density (BV/TV), with the latter parameter being notable in Group 3 (SPTMG). Moreover, the quantitative histomorphometric analysis of neoformed bone area after 8 weeks in the present study also showed the highest values for the perforated mesh groups compared to the occlusive groups, with statistically significant differences between these groups.

In a recent study, the authors compared, in a rat calvarial vertical GBR model, the influence of porosity in a titanium mesh on bone regeneration. The calvaria of nine rats were exposed, and titanium cylinders were set bilaterally. Eighteen surgical sites were randomly allocated into three groups: microporous titanium lid + deproteinized bovine bone mineral (DBBM), macroporous titanium lid + DBBM, and microporous titanium lid + carbonate apatite. The micro-CT evaluation showed an increase in bone volume inside the cylinders in all three groups but without significant differences among the groups [25]. In our study, the qualitative evaluations of the number of trabeculae, trabecular thickness, trabecular separation, and total bone porosity showed no statistically significant differences among the groups, although the numerical values of the groups with porosities in the meshes were the most favorable, especially for Group 3 (SPTMG). This difference could be explained by the microtomographic analysis between the studies (quantitative versus qualitative) and the use of bone substitutes in the bone defects.

Regarding histological evaluations of the influence of membrane and mesh porosities, the literature is more consistent. Klawitter and collaborators studied the bone growth in porous polyethylene rods implanted in dog femurs based on time and pore structure. The results of this investigation have demonstrated that porous polyethylene is capable of accepting bone growth into pores as small as 40 μm. The optimum rate of bone ingrowth was observed in pore sizes of approximately 100 to 135 μm [26]. These results agree with those obtained in the present study, in which the group with a 154.4 μm pore mesh showed a greater area and extent of bone neoformation, with statistically significant differences compared to the groups without porosities in the barriers used.

A recurring finding of the present study was that the use of a PTFE membrane in combination with perforated meshes, creating a more occlusive environment for the cells, resulted in poorer bone neoformation compared to the isolated use of the respective titanium mesh. These results agree with those of Lundgren et al. who, in 1998, evaluated the ability of non-perforated and perforated silicone barriers with different porosities in rat calvarial defects to promote bone tissue augmentation and observed that the use of fully occlusive barriers resulted in the lowest rate of bone augmentation [16].

Regarding the histological percentage of neoformed bone area in the present study, the results of Group 3 (SPTMG) (7.56 ± 1.45%) presented values close to those presented in the study by Yamamoto et al. in rat calvaria for the perforated plastic caps evaluated at 6 weeks (ranging from 4.23% to 13.25%) [13]. Yamamoto et al. evaluated specimens with a later sacrifice time of 12 weeks and observed that in this period the difference in the bone formation in the groups with occlusive and nonocclusive barriers tended to decrease (reaching 25.81% in the totally occlusive group versus 25.09% in the group with 3 perforations in the barrier) [13]. This finding was also reported by other studies [17,25]. The present study had only one evaluation time, 8 weeks, which is a limitation of this study. Perhaps with longer times the results would converge, as described by other studies.

The maintenance of space in the defect area is one of the prerequisites for successful GBR. Therefore, we suggest the use of barriers able to maintain the framework intact without collapsing into the defect or bone substitutes under the barrier if the barrier cannot maintain space on its own. In the present study, there was no filling with bone substitutes, in order not to include an extra factor that could impact the results. The defect was filled only with blood clots in all groups. In this sense, there may have been some negative impact on the bone formation obtained by Group 2 (TEMG), in which the PTFE membrane was not associated with a mesh capable of maintaining the defect volume. It would be interesting to conduct future studies using associated bone grafts, as recently reported in the literature [25,27], to evaluate whether the results obtained here remain.

One possible explanation for the beneficial effect of the presence of porosities in earlier stages of neoformation may be the facilitation of vessel migration into the defects, as shown by the study of Senoo and coworkers (2022), who observed that the blood vessels on the top of the cylinders (barriers used) were smaller in the microporous than in the macroporous groups [25]. An adequate blood supply, angiogenesis, is one of the four pillars of GBR, as described by Wang et al. in 2006 [28]. In the present study, which did not assess vascularization, the greater bone neoformation in the group with microporosity seems to reinforce the importance of this principle.

Recently, new CAD-CAM Titanium meshes individualized by 3D printing have been reported with clinical success in the literature [29]. Among the advantages, manufactured personalized titanium mesh could enable an optimal fit between the mesh and the anatomical shape of the alveolar bone, reconstruct the 3D volume and position of the bone accurately, and shorten the duration of surgery [30]. A recent study carried out the mechanical characterization of 3D-printed individualized Ti mesh [30]. The results showed that the mechanical properties of titanium mesh increased when the thickness decreased (0.5 mm to 0.3 mm) and that with an increase in mesh diameter (3 mm to 5 mm), the mechanical properties of the mesh decreased. Titanium mesh with a thickness of 0.4 mm is strong enough and causes less stimulation to mucosa; therefore, it is more suitable for clinical use. These results agree with those obtained in the present study: the mesh with smaller porosity showed better results, and a thickness of 0.4 mm resulted in good bone formation, with no deformation or fracture of the mesh.

Finally, it is important to emphasize that this study does not propose a change in the concepts of GBR but rather seeks to contribute to the discussion of the possibility of bone neoformation under barriers with different porosities. The comprehension of these phenomena will enable the advancement and refinement of membranes and meshes that are increasingly biocompatible with hard and soft tissues, as well as promote angiogenesis, which is an essential aspect for successful treatment. In fact, recent studies have been progressing in this direction [9,27,31]. Qualitative histomorphometric analyses of the neoformed tissue (such as immunohistochemical evaluations) and controlled clinical studies in humans are needed to further evaluate the biological mechanisms and clinical effectiveness of the concepts discussed here.

## 6. Conclusions

Based on the results of bone neoformation in critical defects in rat calvaria, the following conclusions were reached:

A. Small pore size titanium mesh (154.4 μm) showed the best bone neoformation results, followed by large pore size mesh (850 μm), both qualitatively (higher microtomographic bone density) and quantitatively (larger histomorphometric area and extent of neoformed bone).
B. The association of PTFE membranes with titanium meshes did not improve the outcomes.
C. The use of only the PTFE membrane provided inferior results compared to the use of only titanium meshes.

## Data Availability

The data presented in this study are available on request from the corresponding author. The data are not publicly available due to ethical reasons.

## References

[1] Borie E., Fuentes R., del Sol M., Oporto G., Engelke W. (2011). The influence of FDBA and autogenous bone particles on regeneration of calvaria defects in the rabbit: A pilot study. Ann. Anat.-Anat. Anz..

[2] Atwood D.A. (1971). Reduction of residual ridges: A major oral disease entity. J. Prosthet. Dent..

[3] Araujo M.G., Lindhe J. (2005). Dimensional ridge alterations following tooth extraction. An experimental study in the dog. J. Clin. Periodontol..

[4] Couso-Queiruga E., Stuhr S., Tattan M., Chambrone L., Avila-Ortiz G. (2021). Post-extraction dimensional changes: A systematic review and meta-analysis. J. Clin. Periodontol..

[5] Starch-Jensen T., Becktor J.P. (2019). Maxillary Alveolar Ridge Expansion with Split-Crest Technique Compared with Lateral Ridge Augmentation with Autogenous Bone Block Graft: A Systematic Review. J. Oral Maxillofac. Res..

[6] Elgali I., Omar O., Dahlin C., Thomsen P. (2017). Guided bone regeneration: Materials and biological mechanisms revisited. Eur. J. Oral Sci..

[7] Borges C.D., Faria P.E.P., de Oliveira P.G.F.P., Soares M.S.d.M., Ricoldi M.S.T., Costa M.S., Júnior A.B.N., de Oliveira P.T., Júnior M.T. (2020). Influence of collagen membrane on bone quality in titanium mesh reconstructions—Study in rats. J. Periodontol..

[8] Miyamoto I., Funaki K., Yamauchi K., Kodama T., Takahashi T. (2011). Alveolar Ridge Reconstruction with Titanium Mesh and Autogenous Particulate Bone Graft: Computed Tomography-Based Evaluations of Augmented Bone Quality and Quantity. Clin. Implant. Dent. Relat. Res..

[9] Rakhmatia Y., Ayukawa Y., Furuhashi A., Koyano K. (2014). Microcomputed tomographic and histomorphometric analyses of novel titanium mesh membranes for guided bone regeneration: A study in rat calvarial defects. Int. J. Oral Maxillofac. Implant..

[10] Eisig S.B., Ho V., Kraut R., Lalor P. (2003). Alveolar ridge augmentation using titanium micromesh: An experimental study in dogs. J. Oral Maxillofac. Surg..

[11] Gutta R., Baker R.A., Bartolucci A.A., Louis P.J. (2009). Barrier Membranes Used for Ridge Augmentation: Is There an Optimal Pore Size?. J. Oral Maxillofac. Surg..

[12] Xie Y., Li S., Zhang T., Wang C., Cai X. (2020). Titanium mesh for bone augmentation in oral implantology: Current application and progress. Int. J. Oral Sci..

[13] Yamamoto T., Hasuike A., Koshi R., Ozawa Y., Ozaki M., Kubota T., Sato S. (2018). Influences of mechanical barrier permeability on guided bone augmentation in the rat calvarium. J. Oral Sci..

[14] Ikeno M., Hibi H., Kinoshita K., Hattori H., Ueda M. (2013). Effects of the permeability of shields with autologous bone grafts on bone augmentation. Int. J. Oral Maxillofac. Implant..

[15] Jang Y.-S., Moon S.-H., Nguyen T.-D.T., Lee M.-H., Oh T.-J., Han A.-L., Bae T.-S. (2019). In vivo bone regeneration by differently designed titanium membrane with or without surface treatment: A study in rat calvarial defects. J. Tissue Eng..

[16] Fada R., Shahgholi M., Azimi R., Babadi N.F. (2023). Estimation of Porosity Effect on Mechanical Properties in Calcium Phosphate Cement Reinforced by Strontium Nitrate Nanoparticles: Fabrication and FEM Analysis. Arab. J. Sci. Eng..

[17] Lundgren A.K., Lundgren D., Taylor Å. (1998). Influence of barrier occlusiveness on guided bone augmentation. An experimental study in the rat. Clin. Oral Implant. Res..

[18] Her S., Kang T., Fien M.J. (2012). Titanium Mesh as an Alternative to a Membrane for Ridge Augmentation. J. Oral Maxillofac. Surg..

[19] Kilkenny C., Browne W.J., Cuthill I.C., Emerson M., Altman D.G. (2010). Improving bioscience research reporting: The ARRIVE guidelines for reporting animal research. J. Pharmacol. Pharmacother..

[20] Donath K., Breuner G. (1982). A method for the study of undecalcified bones and teeth with attached soft tissues*. The Sage-Schliff (sawing and grinding) Technique. J. Oral Pathol. Med..

[21] Roccuzzo M., Ramieri G., Bunino M., Berrone S. (2007). Autogenous bone graft alone or associated with titanium mesh for vertical alveolar ridge augmentation: A controlled clinical trial. Clin. Oral Implant. Res..

[22] Proussaefs P., Lozada J. (2006). Use of titanium mesh for staged localized alveolar ridge augmentation: Clinical and histologic-histomorphometric evaluation. J Oral Implantol..

[23] Roccuzzo M., Ramieri G., Spada M.C., Bianchi S.D., Berrone S. (2004). Vertical alveolar ridge augmentation by means of a titanium mesh and autogenous bone grafts. Clin. Oral Implant. Res..

[24] Zellin G., Linde A. (1996). Effects of different osteopromotive membrane porosities on experimental bone neogenesis in rats. Biomaterials.

[25] Senoo M., Hasuike A., Yamamoto T., Ozawa Y., Watanabe N., Furuhata M., Sato S. (2022). Comparison of Macro-and Micro-porosity of a Titanium Mesh for Guided Bone Regeneration: An In Vivo Experimental Study. In Vivo.

[26] Klawitter J.J., Bagwell J.G., Weinstein A.M., Sauer B.W., Pruitt J.R. (1976). An evaluation of bone growth into porous high density polyethylene. J. Biomed. Mater. Res..

[27] Hasegawa H., Masui S., Ishihata H., Kaneko T., Ishida D., Endo M., Kanno C., Yamazaki M., Kitabatake T., Utsunomiya S. (2019). Evaluation of a Newly Designed Microperforated Pure Titanium Membrane for Guided Bone Regeneration. Int. J. Oral Maxillofac. Implant..

[28] Wang H.-L., Boyapati L. (2006). “PASS” Principles for Predictable Bone Regeneration. Implant. Dent..

[29] Ciocca L., Lizio G., Baldissara P., Sambuco A., Scotti R., Corinaldesi G. (2018). Pros-thetically CAD-CAM guided bone augmentation of atrophic jaws using customized titanium mesh: Preliminary results of an open prospective study. J. Oral Implantol..

[30] Bai L., Ji P., Li X., Gao H., Li L., Wang C. (2019). Mechanical Characterization of 3D-Printed Individualized Ti-Mesh (Membrane) for Alveolar Bone Defects. J. Healthc. Eng..

[31] Rakhmatia Y.D., Ayukawa Y., Atsuta I., Furuhashi A., Koyano K. (2014). Fibroblast attachment onto novel titanium mesh membranes for guided bone regeneration. Odontology.

